# Acute renal failure following oxalic acid poisoning: a case report

**DOI:** 10.1186/1745-6673-7-17

**Published:** 2012-09-14

**Authors:** Uditha Dassanayake, Christeine Ariaranee Gnanathasan

**Affiliations:** 1 Department of Clinical Medicine, Faculty of Medicine, University of Colombo, Colombo, Sri Lanka

**Keywords:** Oxalic acid poisoning, Acute renal failure, Sri Lanka

## Abstract

Oxalic acid poisoning is being recognized as an emerging epidemic in the rural communities of Sri Lanka as it is a component of locally produced household laundry detergents. Herein we describe a case of a 32 year old female, presenting after direct ingestion of oxalic acid. She then went on to develop significant metabolic acidosis and acute renal failure, requiring dialysis. Renal biopsy revealed acute tubulointerstitial nephritis associated with diffuse moderate acute tubular damage with refractile crystals in some of the tubules. The patient symptomatically improved with haemodialysis and renal functions subsequently returned to normal.

## Background

Oxalic acid is a toxic organic compound, commonly used as a reducing agent in photography, bleaching and dust removal, as well as being found in certain plants and natural sources. Oxalic acid as a toxin is mostly described in the context of ethylene glycol poisoning, as it is a metabolite of ethylene glycol. Other reported cases of isolated oxalic acid poisoning involve the consumption of food, medications and plants that contain the compound, for example, star fruit and ascorbic acid [1]. Human reports of toxicological effects are relatively uncommon but include gastrointestinal effects, hypocalcemia secondary to calcium oxalate crystal formation and renal toxicity.

Although it is not common in most part of the world, direct intoxication with oxalic acid is a relatively frequent occurrence in some parts of Sri Lanka [2], due to it being a major component of some household laundry detergents. Here we report a case of a patient who went on to develop acute renal failure following self ingestion, and the sequence of events following the event in the local healthcare setting.

### Case Presentation

The patient, a 32 year old female, had ingested approximately 12.5 g of 70% oxalic acid mixed in a glass of water in an attempt at deliberate self harm. Immediately after ingestion, she experienced abdominal pain and profuse vomiting and was brought to the local rural hospital within 2 hours of ingestion. After intravenous access was established and an initial assessment was done, she was immediately transferred to the regional hospital. On arrival, 4 hours after ingestion, she was found to be drowsy but alert (Glasgow coma scale 15), but with a pulse rate (PR) of 50/min and a blood pressure (BP) of 80/60 mmHg. Oxygen saturation on air (SpO_2_) was 92%. Immediate volume resuscitation was done and the patient required inotropic support during the first 24 hours. Vomiting continued throughout the day, with two episodes of blood stained vomitus.

Her urine output was maintained subsequently, but by the 3^rd^ day she developed worsening generalized oedema with a reduction of urine output to approximately 20 ml/hr. There was gradual elevation of serum creatinine (S.Cr) levels (up to 704 umol/dl) and blood urea (BU) levels (up to 43.5 mmol/dl). She was transferred to our hospital on the 3^rd^ day after oxalic acid ingestion. On admission, she was febrile (temperature 99.6°C), nauseous, with stable vital parameters (PR 82/min, BP 120/80 mmHg, SpO_2_ 99% on air) and mild tachypnoea. There was generalized oedema with bilateral fine basal crepitations in the lungs. S.Cr level was 493 umol/dl, with serum potassium (S.K^+^) of 4.8 mEq/l and a white blood cell (WBC) count of 22,500/ul with 87% neutrophils. Urinalysis revealed active sediment (70–80 red cells, 1–2 pus cells/hpf). Arterial blood gases (ABG) showed a metabolic acidosis, with a pH of 7.328, PaCO_2_ of 33.2 mmHg, PaO_2_ of 84.2 mmHg, BE of −6.2 and a HCO_3_ of 18.9 mmol/L.

The patient underwent hemodialysis on the 3^rd^ day, with symptomatic improvement. Post dialysis ABG revealed a respiratory alkalosis (pH of 7.479, PaCO_2_ of 34.9 mmHg, PaO_2_ of 91.9 mmHg, BE of 2.5 and a HCO_3_ of 26.2 mmol/L). The urine output remained low and she required a second dialysis on the 7^th^ day. Urine output increased by day 9, but the S.Cr level remained high.

**Figure 1:**
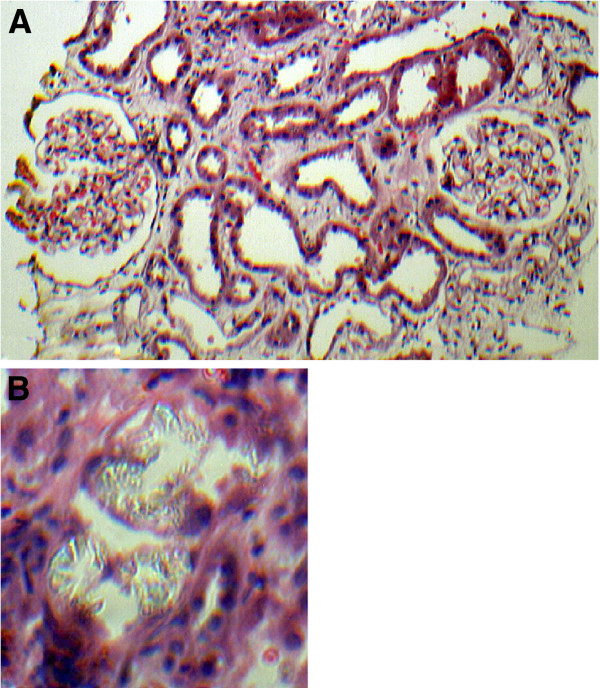
**H & E Staining. A** Necrosis of tubular epithelial cells. Interstitium contains a moderate inflammatory infiltrate composed of neutrophils, lymphocytes and a few eosinophils. Glomeruli are histologically unremarkable. **B** Refractile calcium oxalate crystals within renal tubules.

Renal biopsy was performed on the 8^th^ day revealed acute tubulointerstitial nephritis associated with diffuse moderate acute tubular damage with refractile crystals seen in some tubules (Figure 1). This was reported as being consistent with oxalic acid poisoning. After day 9, the renal functions and urine output gradually improved. No further dialysis was required. She was prescribed antacids and mucoprotective agents and upper gastrointestinal symptoms settled. Blood picture was reported as mild normochromic normocytic anemia (Hb: 9.1 g/dl) with normal WBC and platelets. She was transferred back to the local hospital on 12^th^ day and subsequently discharged on day 14.

On follow up (Day 28), she was asymptomatic with a normal S Cr. Level (114umol/L).

**Figure 2:**
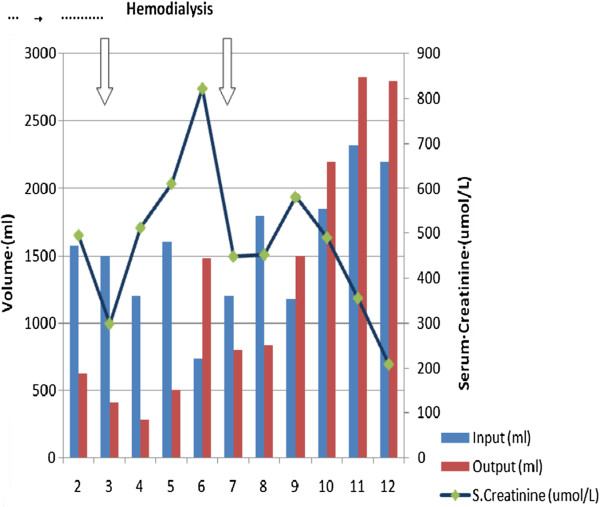
Clinical course – Change in urine output and serum creatinine.

The clinical course of the patient is presented in Figure 2.

### Discussion and conclusions

Self poisoning is a major public health problem in rural Sri Lanka, the majority of the cases being due to ingestion of yellow oleander, paraquat and organophosphorous compounds. There is an estimated prevalence of 315 to 364 per 100,000 population per year attempting self poisoning each year [3,4]. In recent times, the widely used and easily available laundry detergent “Prinso” has been reported to be commonly used in self poisoning attempts, especially in the Southern regions [5]. It is marketed in two sachets, containing 1.2 g of Potassium permanganate (KMNO _4_) and 12.5 g of oxalic acid by many small scale manufacturers and is available under several trade names. Although this case only involves the ingestion of the oxalic acid component, the usual presentation is the simultaneous ingestion of both compounds.

Oxalic acid is poorly absorbed with a bioavailability of 2–5%. It is excreted unchanged in the urine. The oral lethal dose of oxalic acid for adults is 15–30 g, although the ingestion of as little as 5 g has caused death [6]. It may have a direct corrosive effect on the eyes, skin, and digestive tract after contact. However, once absorbed, oxalic acid and other soluble oxalates react with calcium in the plasma to form insoluble calcium oxalate.

Precipitation of calcium oxalate in the renal system (proximal tubules of the kidney) may lead to local necrosis of the tubular epithelium, producing kidney dysfunction and electrolyte imbalance [7]. In renal tubular injury the pathophysiological factors at the cellular level are considered to be energy depletion, cell swelling, calcium influx, intracellular acidosis and enzyme activation [8]. Obstruction of the renal tubules by the crystals is also a mechanism of renal damage. The relative importance of obstruction versus tubular dysfunction is still unclear. The acute renal failure of oxalate poisoning is usually managed supportively, and only a minority of reported cases in Sri Lanka have required dialysis [1].

The renal biopsy specimen revealed associated acute tubulointerstitial nephritis. Interstitial nephritis has been described in cases of chronic hyperoxaluria [9,10]. In this case, the findings are probably attributable to the oxalate itself, although the patient was on the proton pump inhibitor omeprazole, a known cause of interstitial nephritis [11], due to gastrointestinal symptoms.

The neutrophil casts present in some tubules may be attributable to the inflammatory infiltrate often present in acute interstitial nephritis [12], although urinary tract infection could also have been a cause with an indwelling catheter. Urine cultures were negative.

Systemic formation of calcium oxalate may produce hypocalcaemia directly, although the present case had no biochemical or electrocardiographic evidence with a normal serum calcium level recorded on the 2^nd^ day. However, the recorded state of shock with bradycardia at presentation may have been a manifestation of hypocalcaemia, but an electrocardiograph was not available.

In ethylene glycol poisoning, oxalic acid is formed through aldehyde metabolites, and is generally assumed to be the cause of the renal failure associated with ethylene glycol poisoning [13]. The CNS and cardiopulmonary effects are speculated to be due to the aldehyde metabolites. The present case also suggests that the direct toxicity of oxalic acid involves renal failure without significant cardiopulmonary or CNS manifestations.

In conclusion, oxalic acid poisoning has been clearly linked with acute renal impairment, and hence has the potential for fatal consequences. Therefore, better regulation of household products that contain the chemical and education of the general population may be necessary to control this emerging epidemic in the local setting.

### Consent

Written informed consent was obtained from the patient for publication of this Case report and any accompanying images. A copy of the written consent is available for review by the Editor-in-Chief of this journal.

## Competing interests

The authors have no conflicts of interest to declare.

## Author’s contributions

AG overview of patient management, writing up of the case report. UD patient management, writing up of the case report. All authors read and approved the final manuscript.

## Author’s information

AG : MBBS, MPhil, MD, FRCP. Professor in Medicine, Department of Clinical Medicine, Faculty of Medicine, University of Colombo. UD: MBBS. Registrar in Clinical Medicine, University Medical Unit, National Hospital, Colombo.
